# Prevalence and associated factors of napping among older adults in the Philippines

**DOI:** 10.1007/s11325-024-03079-0

**Published:** 2024-08-08

**Authors:** Supa Pengpid, Karl Peltzer

**Affiliations:** 1 Department of Health Education and Behavioral Sciences, Faculty of Public Health, Mahidol University, 420/1 Ratchawithi Road, Ratchathewi, Bangkok, 10400 Thailand; 2 Department of Public Health, Sefako Makgatho Health Sciences University, Pretoria, South Africa; 3 Department of Healthcare Administration, College of Medical and Health Science, Asia University, Taichung, Taiwan; 4 Department of Psychology, University of the Free State, Bloemfontein, South Africa; 5 Department of Psychology, College of Medical and Health Science, Asia University, Taichung, Taiwan

**Keywords:** Sleep duration, Insomnia, Napping, Older adults, Philippines

## Abstract

**Background:**

The aim of the study is to estimate the prevalence and associated factors of insomnia among older adults in the Philippines.

**Methods:**

In all, 5206 cross-sectional nationally representative data from older adults (≥ 60 years) of the 2018 Longitudinal Study on Ageing and Health in the Philippines (LSAHP) was analysed. Napping frequency and duration were assessed by self-report.

**Results:**

The prevalence of regular nappers was 35.7%, low or moderate napping (1–59 min) was 10.5% and long napping (≥ 60 min) duration was 25.2%. In the final adjusted model, older age was not significantly associated with low or moderate napping duration but older age was positively associated with long napping duration. High wealth status, physical activity and late insomnia were positively associated with low or moderate napping duration. High wealth status, urban residence, daily activity limitations, and physical activity were positively associated, and currently working status, poor self-rated health status and current alcohol use were negatively associated with long napping duration.

**Conclusion:**

One in four older adults reported long napping duration. Sociodemographic, health status and behaviour and sleep parameters were associated with low or moderate and/or long napping duration.

## Introduction

For many people, daytime naps are a regular part of their lives [1]. One study among adults in 10 countries found that 23.1% engaged in regular napping, ranging from 42.4% in Brazil to 12.0% in Japan [2]. Biobank data of adults from China reported 21% daytime napping [3, 4] and among adults in rural China, the usual daytime napping was 33.7% [5]. Epidemiology study results indicate that older adults are more likely than younger adults to nap during the day [1]. In USA, in total, 15% of participants indicated they regularly took naps, with the frequency varying from 10% for individuals aged 55–64 to 25% for those aged 75–84 [6], in Japan (≥ 60 years) in men (regular or ≥ 4 times/week) 29.5% and in women 25.7%, and napping ≥ 1 h, 10.5% in men and 7.4% in women [7]. In Australia, 25% of older adults regularly nap [8]. Among middle-aged and older adults in China, 53.4% of individuals reported taking daily afternoon naps [9].

Some reviews found that compared to non-nappers daytime napping can lead to adverse health outcomes, e.g. depression [10], coronary heart disease (CHD) [11], hypertension [12] and obesity [13]. However, some research seems to suggest that short napping (< 30 min) could be beneficial and even more evidence seems to be accumulating that longer napping (≥ 30–60 min) can be detrimental to your health. For example, taking short to moderate naps improved older adults’ cognitive health more than neither not napping nor taking long or prolonged naps [14]. A longer duration of daytime naps (≥ 60 min/day) was linked to an increased risk of cardiovascular disease in comparison to not napping [15]. Additionally, naps during the day that lasted at least one hour had a three times greater impact on the elevated risk of CHD [11] than naps that lasted less than an hour. Napping for ≥ 30 min [16] and ≥ 60 min/day compared to not napping [15] was linked to an increased risk of all-cause death. There was a 35%, 41%, and 40% higher likelihood of cognitive impairment in those who slept for more than 30, 45, and 60 min a day [17]. Prolonged diurnal naps exceeding one hour were linked to a higher chance of incident and prevalent diabetic mellitus [18, 19].

Considering the potential health effects of napping, it would be important to investigate the prevalence and associated factors among older adults, which could enhance the response of health care providers in relation to napping issues [20]. Being scarcity of information on napping in the Philippines [21], this study was trying to fill this gap. Factors associated with regular and/or long napping (≥ 60 min), in particular among older adults, may include sociodemographic factors, health status and behaviour variables and sleep parametres. Sociodemographic factors associated with regular and/or long napping (≥ 60 min) may include older age [3, 4, 7, 22], younger age (≥ 2 h napping) [7], male sex [7, 23–25], higher socioeconomic status (SES) [3], lower neighborhood SES [26], married [3], and residing in rural areas [4].

Variables related to health status that are associated with regular and/or long napping (≥ 60 min) may include poorer self-reported health [25], experiencing bodily pain [6], functional disability [23], and urinary incontinence [6]. Mental health factors may include depression [27], diagnosis of depression [6], higher depression scores [22], psychological distress [7], and cognitive impairment [24]. Health behaviour variables associated with regular and/or long napping (≥ 60 min) may include non-smokers [3], smoking [7, 22, 24], regular drinkers [3], no alcohol use [7], no heavy alcohol use [22], and physical inactivity [3].

Sleep parameters associated with regular and/or long napping (≥ 60 min) may include sleep-maintenance difficulty [28], excessive daytime sleepiness [7, 28], insomnia [7], long sleep [7, 29], shorter sleep duration (napping > 30 min) [30], poor sleep quality [7, 29], use of sleep medication [8], sedating medications [28] and polypharmacy [5]. This study sought to determine the prevalence and correlates of short and long napping in older adults in the Philippines in 2018 as part of a national population survey.

## Methods

### Sample and procedure

The current analyses were conducted using data from the 2018 Longitudinal Study on Ageing and Health in the Philippines (LSAHP) [31]. For the first panel of its implementation, 5985 respondents (60 years of age and older) provided data to the LSAHP, a nationally representative survey. Provinces and villages served as the primary and secondary sampling units, respectively, in a multistage sampling method. Sample restrictions were applied for this study, i.e., responses from proxies were not included. By using this criterion, it was ensured that the responses represented the respondents’ true health status and were provided by them. 5209 respondents made up the analytic sample that was produced. The University of the Philippines Manila Research Ethics Board gave its approval to the LSAHP (MREB 2018-363-01), and participants provided written informed consent [31]. All methods were carried out in accordance with relevant guidelines and regulations and have been performed in accordance with the Declaration of Helsinki.

## Measures

### Outcome measure

Napping was assessed with two questions: (1) “Do you take naps?” (response options were 1 = Yes, regularly, 2 = Yes, not regularly and 3 = No) and (2) “How long do you take naps?” (…Hrs. …minutes). Responses were grouped into (1) non-nappers or not regular nappers, (2) 1–59 min napping of regular nappers and 3) ≥ 60 min napping of regular nappers.

### Exposure variables

*Sociodemographic factors* included age (60–69, 70–79 and 80 or more years), sex, residence status (urban-rural), marital status (never married, currently married, cohabiting, annulled/divorced/separated, widowed), work status (currently working/currently not working) and wealth status. The latter is a wealth index that combines aspects like materials used for the house, facilities present, such as a toilet, and ownership of large items like a vehicle, and grouped into low: 0 = lowest, second, or middle, and high: 1 = fourth or highest.

### Health status and behaviour variables

*Poor self-rated health*. “In general, how would you describe your state of health?” (Coded 1=”somewhat unhealthy or very unhealthy”, and 0=”very healthy, healthier than average or of average health”).

*Body pain*. “Are you often troubled with pain?” (Yes/No).

*Depressive symptoms* were assessed with the 11-item 3-response category CES-D Scale [32], using a cut-off score of 7 or more [33] (Cronbach’s alpha was 0.74 in this study).

*Loneliness* (scores 6–9 = 1, and 1–5 = 0) was assessed with the three-item subscale from the revised UCLA loneliness scale [34] (Cronbach’s alpha was 0.83 in this study).

*Loss of bladder control*. “Do you experience loss of bladder control?” (Yes/No).

*Impaired cognition* (scores ≥ 8) was assessed with the Telephone Interview for Cognitive Status (TICS-HRS-35), including orientation questions, counting backwards, object naming, serial 7s subtraction, and 10-word immediate and delayed recall (score range 0–35) [35].

*Activities of daily living (ADL) limitations* (defined as any one of seven ADLs due to health reasons), e.g., “dressing”, and “using the toilet” [31] (Cronbach’s alpha was 0.82 in this study).

*Physical activity.* “How often do you engage in physical exercises such as walking, calisthenics, ballroom dancing?” (1(Low)=”never, a few times a year, or about once a month”, 2(Moderate)=”about once a week, or several times a week”, and 3(High)=”every day”).

*Current smoking*. “Do you currently smoke cigarettes/cigar?” (Yes/No).

*Current alcohol use*. “Do you currently drink alcohol?” (Yes/No) [31]. .

### Sleep parameters

*Initial insomnia* was defined as “most of the time having trouble falling asleep.”

*Intermediate insomnia* was defined as “most of the time having trouble with waking up during the night.”

*Late insomnia* was defined as “most of the time having trouble with waking up too early and not being able to fall asleep again.”

*Daytime sleepiness* was defined as “never feeling really rested when you wake up in the morning.” [36].

*Sleep quality* was assessed with the question, “Are you satisfied with your sleep?” ) (Yes/No) A “no” response was defined as poor sleep quality.

*Nocturnal sleep duration* was assessed with the question, “On average, approximately how much do you sleep per night?” (…Hrs. …Mins.) and grouped into < 6 h, 6–6.9 h, 7–8.9 h and 9 or more hours.

*Use of sleep medication* was sourced from the item, “In the past two weeks, have you taken any medications or used other treatments to help you sleep?” (Yes/No).

### Data analysis

Descriptive statistics were used to describe the sample and napping categories. Chi-square statistics were used to test for differences in proportions. Using multinomial logistic regression, associations between social and health variables and napping duration categories were determined, overall and stratified by working status. Crude (unadjusted) associations are the estimated association between exposure and outcome before potential confounding variables are taken into account. Adjusted associations are multivariable models that show the relationship between multiple characteristics and the outcome while accounting for the impact of other characteristics. Based on an earlier review of the literature, covariates were added. Covariates significant (*p* < 0.5) in univariable analyses were subsequently included in the multivariable models. In the overall model, covariates included age, wealth status, urban residence, work status, poor self-rated health status, body pain, depressive symptoms, impaired cognition, ADL limitations, physical activity, current smoking, current alcohol use and insomnia symptoms. In the second model with the working sample, covariates included, wealth status, poor self-rated health status, depressive symptoms, impaired cognition, physical activity, current smoking, insomnia symptoms and use of sleep medication. In the third model with the non-working sample, covariates included, age, sex, wealth status, urban residence, poor self-rated health status, body pain, depressive symptoms, ADL limitations, physical activity, current alcohol use, insomnia symptoms and nocturnal sleep duration.

Weights were applied to both regression analyses and descriptive statistics to account for the intricate sampling design [31]. Variance Inflation Factor (VIF) did not show any collinearity. At *p* < 0.05, all tests were deemed statistically significant. For all statistical procedures, STATA software (Stata Corporation, College Station, TX, USA) version 15.0 was utilized.

## Results

The analytic sample included 5209 participants (median age 66 years, IQR = 9), including 40.2% men. The prevalence of regular nappers was 35.7%, low or moderate napping (1–59 min) was 10.5% and long napping (≥ 60 min) duration was 25.2%. Napping status differed by age group, wealth, work, widowhood and residence status, sex, body pain, impaired cognition, depressive symptoms, loneliness, physical activity level, ADL limitations, current alcohol use, initial, intermediate, and late insomnia symptoms, poor sleep quality and nocturnal sleep duration (see Table 1).

**Table 1:** Sample and napping characteristics, Philippines, 2018

Variable	Sample	Napping in minutes of regular nappers			
		0	1–59	≥ 60	*p*-value^a^
	N (%)	N (%)	N (%)	N (%)	
**All**	5209	3240 (64.3)	543 (10.5)	1426 (25.2)	
**Sociodemographic factors**					
Age (in years)					
60–69 70–79 80 or more	2075 (66.3) 2122 (25.7) 1012 (8.1)	1378 (68.7) 1316 (58.1) 546 (49.6)	212 (11.0) 212 (8.6) 119 (13.2)	485 (20.3) 594 (33.3) 347 (37.2)	< 0.001
Sex					
Female Male	3301 (59.8) 1908 (40.2)	2062 (64.5) 1178 (63.9)	373 (10.3) 170 (10.7)	866 (25.1) 560 (25.3)	0.004
Wealth status					
Low/middle High	3353 (65.4) 1856 (34.6)	2262 (73.2) 978 (46.7)	278 (6.5) 265 (18.4)	813 (20.3) 613 (34.9)	< 0.001
Residence status					
Rural Urban	2913 (56.8) 2296 (43.2)	2027 (72.8) 1213 (52.5)	218 (8.3) 325 (13.6)	668 (18.9) 758 (33.9)	< 0.001
Widowed	2526 (39.7)	1506 (63.1)	286 (9.5)	734 (27.4)	< 0.001
Working	1853 (48.8)	1295 (74.9)	161 (8.2)	397 (16.9)	< 0.001
**Health status and behaviour**					
Poor self-rated health	1905 (30.2)	1183 (70.3)	210 (9.2)	512 (20.5)	0.491
Body pain	1796 (33.4)	1146 (71.0)	201 (9.7)	449 (19.3)	0.015
Depressive symptoms	1868 (32.1)	1210 (70.9)	208 (8.3)	450 (20.8)	< 0.001
Loneliness	797 (14.4)	470 (63.0)	104 (9.9)	223 (27.1)	0.019
Loss of bladder control	646 (10.6)	379 (62.0)	68 (11.2)	543 (26.9)	0.098
Impaired cognition	431 (6.8)	258 (72.0)	32 (5.7)	141 (22.4)	0.009
ADL limitations	985 (18.7)	547 (58.0)	111 (11.0)	327 (31.1)	< 0.001
Physical activity					
Low Moderate High	1326 (23.8) 1130 (21.7) 2753 (54.4)	880 (80.0) 694 (54.5) 1666 (60.5)	99 (4.1) 130 (14.3) 314 (12.2)	347 (15.9) 306 (31.2) 773 (27.3)	< 0.001
Current smoking	707 (17.3)	437 (72.1)	65 (5.0)	205 (22.9)	0.369
Current alcohol use	1393 (29.7)	943 (75.7)	111 (7.3)	339 (17.1)	< 0.001
**Sleep parameters**					
Initial insomnia	802 (15.2)	500 (64.7)	112 (13.9)	189 (21.4)	< 0.001
Intermediate insomnia	859 (14.7)	508 (63.7)	118 (11.5)	233 (24.8)	0.002
Late insomnia	1306 (25.0)	781 (63.5)	183 (15.4)	342 (21.1)	< 0.001
Daytime sleepiness	190 (2.9)	125 (73.9)	22 (7.2)	43 (19.0)	0.320
Poor sleep quality	957 (17.4)	636 (66.4)	111 (11.4)	210 (22.2)	< 0.001
Nocturnal sleep duration (in hrs)					
7-8.9 <6 6-6.9 9 or more	1840 (36.6) 1812 (32.7) 1204 (24.7) 344 (6.1)	1156 (65.0) 1130 (65.5) 755 (61.4) 192 (62.9)	177 (11.7) 208 (9.8) 128 (11.2) 30 (4.2)	507 (23.2) 474 (24.7) 321 (27.4) 122 (32.9)	0.014
Sleep medication	93 (1.0)	59 (74.1)	10 (5.6)	24 (20.4)	0.943

ADL = Activities of Daily Living; ^a^Chi-square statistics

### Crude associations with low or moderate and long napping duration

Older age, high wealth status, urban residence, physical activity, and late insomnia symptoms were positively associated with low or moderate napping duration. Currently working, impaired cognition, depressive symptoms and current smoking were negatively associated with low or moderate napping duration. Older age, high wealth status, urban residence, physical activity, and ADL limitations were positively associated with long napping duration. Currently working, poor self-rated health status, body pain, depressive symptoms, and current alcohol use were negatively associated with long napping duration (see Table 2).

**Table 2:** Crude associations with low or moderate and long napping duration (with no napping as reference category) estimated by multinomial logistic regression

Variables	Napping 1–59 min		Napping ≥ 60 min	
	CRRR (95% CI)	*p*-value	CRRR (95% CI)	*p*-value
**Sociodemographic factors**				
Age (in years)				
60–69	1 (Reference)		1 (Reference)	
70–79 80 or more	0.80 (0.40 to 1.58) 1.71 (1.07 to 2.75)	0.513 0.026	1.81 (0.91 to 3.59) 3.16 (2.34 to 4.26)	0.091 < 0.001
Sex (male)	1.05 (0.57 to 1.96)	0.865	1.09 (0.83 to 1.44)	0.520
Wealth status (High)	4.57 (2.48 to 8.43)	< 0.001	2.57 (1.72 to 3.86)	< 0.001
Urban residence	2.37 (1.36 to 4.14)	0.002	2.40 (1.62 to 3.56)	< 0.001
Widowed	0.96 (0.58 to 1.57)	0.859	1.35 (0.98 to 1.87)	0.070
Working	0.48 (0.25 to 0.92)	0.026	0.36 (0.23 to 0.55)	< 0.001
**Health status and behaviour**				
Poor self-rated health status	0.72 (0.37 to 1.40)	0.335	0.66 (0.47 to 0.92)	0.015
Body pain	0.75 (0.48 to 1.17)	0.207	0.58 (0.39 to 0.86)	0.008
Depressive symptoms	0.61 (0.39 to 0.94)	0.026	0.65 (0.43 to 0.97)	0.034
Loneliness	0.95 (0.60 to 1.53)	0.845	1.11 (0.69 to 1.81)	0.659
Loss of bladder control	0.87 (0.38 to 1.98)	0.733	0.90 (0.49 to 1.63)	0.716
Impaired cognition	0.45 (0.21 to 0.99)	0.047	0.78 (0.48 to 1.27)	0.320
ADL limitations	1.31 (0.83 to 2.09)	0.246	1.86 (1.36 to 2.54)	< 0.001
Physical activity				
Low	1 (Reference)		1 (Reference)	
Moderate High	4.91 (2.05 to 11.76) 3.66 (1.84 to 7.30)	< 0.001 < 0.001	2.12 (1.16 to 3.88) 1.72 (0.96 to 3.08)	0.015 0.068
Current smoking	0.43 (0.22 to 0.86)	0.018	0.82 (0.47 to 1.43)	0.477
Current alcohol use	0.51 (0.25 to 1.05)	0.068	0.54 (0.34 to 0.84)	0.007
**Sleep parameters**				
Initial insomnia	1.39 (0.87 to 2.22)	0.171	0.82 (0.52 to 1.31)	0.406
Intermediate insomnia	1.13 (0.72 to 1.80)	0.593	0.99 (0.66 to 1.49)	0.964
Late insomnia	1.75 (1.23 to 2.48)	0.002	0.80 (0.51 to 1.28)	0.361
Daytime sleepiness	0.61 (0.27 to 1.37)	0.226	0.64 (0.34 to 1.21)	0.167
Poor sleep quality	1.07 (0.62 to 1.85)	0.814	0.82 (0.55 to 1.23)	0.339
Nocturnal sleep duration (in hours)				
7-8.9	1 (Reference)		1 (Reference)	
<6 6-6.9 9 or more	0.83 (0.48 to 1.45) 1.01 (0.41 to 2.50) 0.38 (0.14 to 1.06)	0.515 0.976 0.064	1.06 (0.70 to 1.59) 1.25 (0.80 to 1.96) 1.47 (0.94 to 2.32)	0.792 0.329 0.094
Sleep medication	0.72 (0.33 to 1.59)	0.413	0.88 (0.46 to 1.67)	0.690

CRRR = Crude Relative Risk Ratio; CI = Confidence interval; ADL = Activities of Daily Living

### Adjusted associations with low or moderate and long napping duration

In the final adjusted model, older age was not significantly associated with low or moderate (short) napping duration but older age was positively associated with long napping duration. High wealth status, physical activity and late insomnia increased the likelihood of short napping duration. Having a high wealth status, urban residence, ADL limitations, and physical activity increased the risk ratio of long napping duration. Currently working status, poor self-rated health status and current alcohol use decreased the likelihood of long napping duration (see Table 3).

**Table 3:** Adjusted associations with low or moderate and long napping duration (with no napping as reference category)

Variables	Napping 1–59 min		Napping ≥ 60 min	
	ARRR (95% CI)	*p*-value	ARRR (95% CI)	*p*-value
**Sociodemographic factors**				
Age (in years)				
60–69	1 (Reference)		1 (Reference)	
70–79 80 or more	0.92 (0.64 to 1.33) 1.45 (0.84 to 2.54)	0.664 0.183	1.94 (1.29 to 2.91) 2.27 (1.60 to 3.24)	0.002 < 0.001
Wealth status (High)	3.74 (2.00 to 6.97)	< 0.001	2.03 (1.37 to 3.00)	< 0.001
Urban residence	1.36 (0.70 to 2.64)	0.355	1.65 (1.20 to 2.29)	0.003
Working	0.63 (0.32 to 1.24)	0.178	0.54 (0.36 to 0.82)	0.003
**Health status and behaviour**				
Poor self-rated health status	0.86 (0.43 to 1.72)	0.661	0.69 (0.52 to 0.93)	0.015
Body pain	1.06 (0.65 to 1.74)	0.807	0.76 (0.54 to 1.08)	0.123
Depressive symptoms	0.67 (0.44 to 1.03)	0.066	0.80 (0.55 to 1.19)	0.278
Impaired cognition	0.49 (0.21 to 1.16)	0.105	0.71 (0.47 to 1.07)	0.098
ADL limitations	1.35 (0.80 to 2.27)	0.256	1.53 (1.13 to 2.08)	0.007
Physical activity				
Low	1 (Reference)		1 (Reference)	
Moderate High	5.25 (2.48 to 11.13) 3.58 (1.92 to 6.62)	< 0.001 < 0.001	3.03 (1.85 to 4.97) 1.93 (1.22 to 3.06)	< 0.001 0.005
Current smoking	0.60 (0.27 to 1.38)	0.228	1.40 (0.84 to 2.32)	0.195
Current alcohol use	0.67 (0.32 to 1.39)	0.277	0.61 (0.40 to 0.92)	0.020
**Insomnia symptoms**				
Initial insomnia	1.34 (0.78 to 2.31)	0.284	0.94 (0.52 to 1.70)	0.844
Intermediate insomnia	0.70 (0.39 to 1.28)	0.246	1.15 (0.71 to 1.88)	0.564
Late insomnia	2.29 (1.49 to 3.52)	< 0.001	0.96 (0.58 to 1.59)	0.874
Daytime sleepiness	0.61 (0.25 to 1.47)	0.226	0.57 (0.32 to 1.01)	0.056

ARRR = Adjusted Relative Risk Ratio; CI = Confidence interval; ADL = Activities of Daily Living

### Crude associations with low or moderate and long napping duration among those currently working

High wealth status, physical activity and late insomnia symptoms were positively associated and poor self-rated health status, impaired cognition, and current smoking were negatively associated with low or moderate napping duration. High wealth status, and physical activity were positively associated and depressive symptoms, impaired cognition and intake of sleep medication was negatively associated with long napping duration (see Table 4).

**Table 4:** Crude associations with low or moderate and long napping duration (with no napping as reference category) among working sample

Variables	Napping 1–59 min		Napping ≥ 60 min	
	CRRR (95% CI)	*p*-value	CRRR (95% CI)	*p*-value
**Sociodemographic factors**				
Age (in years)				
60–69	1 (Reference)		1 (Reference)	
70–79 80 or more	0.46 (0.17 to 1.25) 2.91 (0.81 to 10.48)	0.126 0.103	1.24 (0.54 to 2.83) 1.52 (0.76 to 3.03)	0.616 0.230
Sex (male)	0.62 (0.25 to 1.53)	0.293	1.48 (0.84 to 2.60)	0.171
Wealth status (High)	5.58 (2.18 to 14.32)	< 0.001	1.95 (1.19 to 3.20)	0.009
Urban residence	2.39 (0.79 to 7.21)	0.120	1.83 (0.94 to 3.57)	0.074
Widowed	0.87 (0.42 to 1.80)	0.709	1.00 (0.58 to 1.72)	0.998
**Health status and behaviour**				
Poor self-rated health status	0.32 (0.12 to 0.83)	0.020	0.50 (0.25 to 1.00)	0.050
Body pain	0.52 (0.22 to 1.25)	0.144	0.53 (0.28 to 1.03)	0.061
Depressive symptoms	0.55 (0.24 to 1.27)	0.161	0.45 (0.22 to 0.92)	0.028
Loneliness	1.06 (0.43 to 2.58)	0.906	0.78 (0.32 to 1.90)	0.587
Loss of bladder control	1.75 (0.66 to 4.65)	0.258	0.70 (0.34 to 1.34)	0.326
Impaired cognition	0.18 (0.05 to 0.72)	0.016	0.30 (0.11 to 0.83)	0.020
ADL limitations	1.28 (0.44 to 3.69)	0.643	0.66 (0.27 to 1.58)	0.343
Physical activity				
Low	1 (Reference)		1 (Reference)	
Moderate High	11.23 (2.87 to 44.01) 4.63 (1.93 to 11.08)	< 0.001 < 0.001	3.99 (1.95 to 8.17) 2.55 (1.31 to 4.98)	< 0.001 0.006
Current smoking	0.26 (0.09 to 0.71)	0.009	1.17 (0.52 to 2.67)	0.699
Current alcohol use	0.87 (0.27 to 2.75)	0.807	0.91 (0.45 to 1.87)	0.802
**Sleep parameters**				
Initial insomnia	1.53 (0.61 to 3.81)	0.361	0.76 (0.34 to 1.68)	0.488
Intermediate insomnia	1.31 (0.57 to 3.01)	0.524	1.74 (0.81 to 3.70)	0.152
Late insomnia	2.44 (1.39 to 4.31)	0.002	1.17 (0.57 to 2.40)	0.676
Daytime sleepiness	0.40 (0.08 to 1.93)	0.252	1.25 (0.41 to 3.86)	0.692
Poor sleep quality	1.23 (0.47 to 3.20)	0.669	1.05 (0.49 to 2.23)	0.906
Nocturnal sleep duration (in hours)				
7-8.9	1 (Reference)		1 (Reference)	
<6 6-6.9 9 or more	0.89 (0.45 to 1.74) 1.76 (0.45 to 6.96) 0.56 (0.14 to 2.25)	0.726 0.415 0.407	1.07 (0.55 to 2.07) 1.38 (0.70 to 2.72) 1.22 (0.47 to 3.16)	0.846 0.344 0.679
Sleep medication	0.25 (0.04 to 1.44)	0.121	0.12 (0.02 to 0.65)	0.014

CRRR = Crude Relative Risk Ratio; CI = Confidence interval; ADL = Activities of Daily Living

### Adjusted associations with low or moderate and long napping duration among those currently working

In the final adjusted model, among those currently working, high wealth status, physical activity and late insomnia increased the likelihood of short napping duration, while poor self-rated health status, current smoking and intake of sleep medication decreased the likelihood of short napping duration. Physical activity increased the likelihood, and depressive symptoms, and intake of sleep medication decreased the likelihood of long napping duration among those who were working (see Table 5).

**Table 5:** Adjusted associations with low or moderate and long napping duration (with no napping as reference category) among working sample

Variables	Napping 1–59 min		Napping ≥ 60 min	
	ARRR (95% CI)	*p*-value	ARRR (95% CI)	*p*-value
**Sociodemographic factors**				
Wealth status (High)	4.98 (2.20 to 11.25)	< 0.001	1.64 (0.97 to 2.76)	0.063
**Health status and behaviour**				
Poor self-rated health status	0.37 (0.15 to 0.92)	0.032	0.64 (0.35 to 1.20)	0.163
Depressive symptoms	0.93 (0.54 to 1.59)	0.777	0.53 (0.29 to 0.95)	0.032
Impaired cognition	0.27 (0.05 to 1.58)	0.146	0.37 (0.13 to 1,03)	0.058
Physical activity				
Low	1 (Reference)		1 (Reference)	
Moderate High	8.35 (2.74 to 25.39) 3.73 (1.56 to 8.90)	< 0.001 0.003	3.88 (2.09 to 7.18) 2.16 (1.21 to 3.84)	< 0.001 0.009
Current smoking	0.30 (0.13 to 0.71)	0.006	1.34 (0.73 to 2.45)	0.341
**Sleep parameters**				
Initial insomnia	1.27 (0.47 to 3.39)	0.633	0.68 (0.26 to 1.77)	0.424
Intermediate insomnia	0.76 (0.29 to 1.98)	0.576	2.19 (1.00 to 4.80)	0.051
Late insomnia	3.51 (1.90 to 6.51)	< 0.001	1.23 (0.59 to 2.56)	0.676
Sleep medication	0.12 (0.02 to 0.84)	0.033	0.11 (0.02 to 0.67)	0.016

ARRR = Adjusted Relative Risk Ratio; CI = Confidence interval

### Crude associations with low or moderate and long napping duration among those currently not working

Male sex, high wealth status, physical activity, and urban residence were positively associated, and depressive symptoms, current alcohol use, and 9 or more hours nocturnal sleep duration were negatively associated with low or moderate napping duration. Older age, high wealth status, urban residence, and ADL limitations were positively associated, and poor self-rated health status, body pain, depressive symptoms, late insomnia and daytime sleepiness were negatively associated with long napping duration (see Table 6).

**Table 6:** Crude associations with low or moderate and long napping duration (with no napping as reference category) among non-working sample

Variables	Napping 1–59 min		Napping ≥ 60 min	
	CRRR (95% CI)	*p*-value	CRRR (95% CI)	*p*-value
**Sociodemographic factors**				
Age (in years)				
60–69	1 (Reference)		1 (Reference)	
70–79 80 or more	0.72 (0.36 to 1.42) 1.09 (0.60 to 2.01)	0.342 0.769	1.57 (0.83 to 2.96) 2.19 (1.53 to 3.14)	0.167 < 0.001
Sex (male)	2.08 (1.03 to 4.22)	0.042	1.47 (0.91 to 2.36)	0.114
Wealth status (High)	3.73 (2.03 to 6.89)	< 0.001	2.57 (1.63 to 4.05)	< 0.001
Urban residence	2.14 (1.09 to 4.24)	0.028	2.39 (1.33 to 4.28)	0.004
Widowed	0.87 (0.47 to 1.29)	0.638	1.27 (0.84 to 1.93)	0.257
**Health status and behaviour**				
Poor self-rated health status	1.00 (0.50 to 2.00)	0.990	0.70 (0.53 to 0.90)	0.007
Body pain	1.08 (0.64 to 1.82)	0.761	0.71 (0.52 to 0.95)	0.023
Depressive symptoms	0.61 (0.37 to 0.99)	0.046	0.71 (0.51 to 0.97)	0.035
Loneliness	0.77 (0.45 to 1.31)	0.329	1.03 (0.64 to 1.64)	0.915
Loss of bladder control	0.49 (0.15 to 1.56)	0.228	0.73 (0.28 to 1.95)	0.531
Impaired cognition	0.51 (0.20 to 1.27)	0.145	0.83 (0.52 to 1.31)	0.419
ADL limitations	1.17 (0.69 to 1.98)	0.568	2.05 (1.48 to 2.84)	< 0.001
Physical activity				
Low	1 (Reference)		1 (Reference)	
Moderate High	2.93 (1.45 to 5.92) 3.41 (1.45 to 8.04)	0.003 0.005	1.67 (0.79 to 3.53) 1.53 (0.74 to 3.20)	0.181 0.251
Current smoking	0.86 (0.36 to 2.05)	0.737	0.96 (0.44 to 2.06)	0.909
Current alcohol use	0.36 (0.17 to 0.76)	0.007	0.51 (0.26 to 1.11)	0.052
**Sleep parameters**				
Initial insomnia	1.11 (0.65 to 1.89)	0.708	0.68 (0.40 to 1.16)	0.155
Intermediate insomnia	0.88 (0.51 to 1.51)	0.644	0.60 (0.36 to 1.01)	0.055
Late insomnia	1.30 (0.82 to 2.07)	0.267	0.61 (0.38 to 0.97)	0.038
Daytime sleepiness	0.58 (0.23 to 1.48)	0.255	0.36 (0.16 to 0.83)	0.016
Poor sleep quality	0.93 (0.52 to 1.67)	0.813	0.69 (0.43 to 1.09)	0.110
Nocturnal sleep duration (in hours)				
7-8.9	1 (Reference)		1 (Reference)	
<6 6-6.9 9 or more	0.76 (0.40 to 1.44) 0.63 (0.28 to 1.44) 0.24 (0.06 to 0.99)	0.396 0.272 0.048	0.99 (0.63 to 1.54) 1.14 (0.72 to 1.79) 1.10 (0.73 to 1.66)	0.963 0.577 0.655
Sleep medication	0.69 (0.29 to 1.59)	0.381	0.80 (0.39 to 1.64)	0.540

CRRR = Crude Relative Risk Ratio; CI = Confidence interval; ADL = Activities of Daily Living

### Adjusted associations with low or moderate and long napping duration among those currently not working

In the final adjusted model, among those currently not working, male sex, high wealth status, and physical activity increased the likelihood of short napping duration, and current alcohol use decreased the likelihood of short napping duration. Older, age, male sex, high wealth status, urban residence, ADL limitations, and moderate physical activity increased the risk ratio and current alcohol use decreased risk ratio of long napping duration (see Table 7).

**Table 7:** Adjusted associations with low or moderate and long napping duration (with no napping as reference category) among non-working sample

Variables	Napping 1–59 min		Napping ≥ 60 min	
	ARRR (95% CI)	*p*-value	ARRR (95% CI)	*p*-value
**Sociodemographic factors**				
Age (in years)				
60–69	1 (Reference)		1 (Reference)	
70–79 80 or more	0.95 (0.56 to 1.61) 1.06 (0.57 to 1.98)	0.840 0.845	2.01 (1.23 to 3.30) 2.03 (1.37 to 2.98)	0.006 < 0.001
Sex (male)	2.58 (1.15 to 5.77)	0.021	1.63 (1.09 to 2.44)	0.018
Wealth status (High)	2.94 (1.81 to 4.76)	< 0.001	2.07 (1.41 to 3.04	< 0.001
Urban residence	1.25 (0.64 to 2.43)	0.511	1.69 (1.02 to 2.80)	0.040
**Health status and behaviour**				
Poor self-rated health status	1.17 (0.54 to 2.53)	0.690	0.78 (0.58 to 1.04)	0.094
Body pain	1.40 (0.80 to 2.44)	0.236	0.85 (0.58 to 1.25)	0.409
Depressive symptoms	0.69 (0.44 to 1.09)	0.111	0.92 (0.65 to 1.30)	0.630
ADL limitations	1.16 (0.68 to 1.98)	0.570	1.83 (1.26 to 2.66)	0.002
Physical activity				
Low	1 (Reference)		1 (Reference)	
Moderate High	2.48 (1.32 to 4.62) 2.89 (1.38 to 6.04)	0.005 0.005	1.97 (1.09 to 3.57) 1.66 (0.97 to 2.83)	0.025 0.064
Current alcohol use	0.38 (0.20 to 0.71)	0.003	0.52 (0.28 to 0.98)	0.044
**Sleep parameters**				
Initial insomnia	1.39 (0.75 to 2.58)	0.291	1.23 (0.60 to 2.51)	0.568
Intermediate insomnia	0.74 (0.39 to 1.41)	0.361	0.76 (0.41 to 1.44)	0.403
Late insomnia	1.61 (0.91 to 2.85)	0.100	0.71 (0.40 to 1.26)	0.242
Nocturnal sleep duration (in hours)				
7-8.9	1 (Reference)		1 (Reference)	
<6 6-6.9 9 or more	0.88 (0.52 to 1.48) 0.64 (0.30 to 1.46) 0.40 (0.12 to 1.33)	0.625 0.243 0.132	1.33 (0.82 to 2.15) 1.14 (0.73 to 1.79) 1.50 (0.81 to 2.79)	0.246 0.559 0.198

ARRR = Adjusted Relative Risk Ratio; CI = Confidence interval; ADL = Activities of Daily Living

## Discussion

According to the study, the prevalence of regular nappers (35.7%), low or moderate nappers (1–59 min) (10.5%) and long nappers (≥ 60 min) (25.2%) of older adults in the Philippines (those aged 60 and above) in a large national sample in 2018, was higher than among older adults (≥ 60 years) in Japan (regular, ≥ 4 times/week, 29.5% in women 25.7% in men) [7], napping ≥ 1 h (10.5% in men and 7.4% in women) [7], in USA (≥ 55 years, regular napping 15%) [6], Australia (older adults, regular napping 25%) [8] and lower than in China (middle-aged and older adults, daily afternoon naps 53.4%) [9]. Some of these differences may also be attributed to differences in measurement of habitual nappers.

Major results of the current study indicated that some of the same factors were linked to both short and long nap times, including high wealth status, and physical activity. Factors uniquely associated with short napping included late insomnia, and uniquely associated with long napping included older age, urban residence, not working status, ADL limitations, not poor self-rated health status, and not current alcohol use. Comparing the working population with the non-working population, older age, urban residence, and ADL limitations were uniquely associated with long napping and male sex and non-current alcohol use with short and long napping in the non-working population. Not having a poor self-rated health status, non-current smoking, and late insomnia were uniquely associated with short napping in the working population. In addition, non-sleep medication use was uniquely associated with short and long napping and not having depressive symptoms with long napping in the working population.

Regarding sociodemographic factors associated with regular and/or long napping (≥ 60 min), the study found, in agreement with some previous research [4, 7, 22, 24, 29] that long napping increased with age in the overall sample, and among those not working but not among those who were working, while the frequency of short napping did not differ by age. This could mean that when older adults stop working or retire, they engage more likely in long napping. The higher prevalence of long napping in older age groups and when older adults stop working may be included in the sleep management of older adults in the Philippines. Consistent with several research findings [3, 7, 23–25], male sex in the non-working sample and higher wealth status were positively associated with low or moderate and long napping duration. Understandingly, working status was negatively associated with long napping duration. Further, the study found that urban residence was positively associated with long napping, while among adults in China daytime napping habit was higher in rural areas [4]. We did not discover any significant correlation between widowhood and either long or short naps, despite a Chinese study among adults finding an association between being married [3].

Regarding health status, this study found in agreement with previous research [23, 25] that poor self-rated health and having ADL limitations increased the risk ratio of long napping. A study among adults in Japan found a positive association between psychological distress and napping [7], while in our study, among those who were working, depressive symptoms was negatively associated with long moderate napping duration. Depressed older adults may feel insecure in their social environment hindering them to engage in napping [37]. In a study among older adults in USA, experiencing bodily pain [6] was associated with napping, while this study showed in unadjusted analysis a negative association between body pain and long napping duration. Unlike several previous studies [3, 6, 22, 27], this survey did not find a significant correlation between loneliness, urinary incontinence, cognitive impairment and short or long napping.

In terms of health risk behaviour, some previous research showed mixed results in relation to smoking status, e.g., non-smokers [3] and current smoking [7, 22, 24] was associated with napping, while this study found among the working sample that current smoking was negatively associated with low or moderate napping. Similarly, mixed results having been reported on alcohol use, e.g., regular drinkers [3] and no alcohol use [7] and no heavy alcohol use [22] being associated with napping, while this study found that current alcohol use decreased the risk ratio of long napping, in particular, in the non-working sample. It is possible that as long napping increases with age, alcohol use decreases with age, and/or older adults may refrain from drinking alcohol in anticipation of its potentially negative effects on sleep [7]. Finally, a previous study in China found positive association between physical inactivity and napping [3], and another study in China found that higher physical functioning was associated with short (≤ 60 min) and not long (> 60 min) napping [38], while this study found a positive association between physical activity and both short and long napping. In a study among older adults in Brazil [39], walking regularly was found to have a significant correlation with short-duration daytime naps, while our study also found among the non-working sample that physical activity was associated with short but not long napping. A systematic review showed that day-time napping improved short-term physical performance [40].

Concerning sleep parameters, previous research found a positive association between sleep-maintenance difficulty [28], and insomnia [7] with napping, while this study found that late insomnia was positively associated with low or moderate napping duration, particularly in the working sample. This shows that nocturnal sleep deficit may translate in an increase of short daytime napping. Regarding sleep duration, previous research found a positive association between long sleep [7, 29], and shorter nocturnal sleep duration (napping > 30 min) [30] and napping, while this study found that short and long nocturnal sleep duration was not significantly associated with low or moderate napping duration, only in unadjusted analysis among the non-working sample long nocturnal sleep duration was negatively associated with short napping. It may be plausible that long nocturnal sleep duration is associated with lower short napping, having a lessor need to compensate for shorter nocturnal sleep [5]. Contrary to some previous studies [7, 28] that found a positive association between daytime sleepiness and frequent and long napping, this study found no association between daytime sleepiness and napping. This may be explained by the overall very low (2.9%) prevalence of daytime sleepiness in this study and consequently not confirming that napping can be caused by daytime sleepiness. This finding may suggest that napping in the Philippines is a cultural habit, unlike the US population, where taking naps during the day is typically a result of exhaustion and sleepiness during the day rather than a cultural habit [26]. Unlike previous studies [8, 29], this study did not find a significant association between poor sleep quality and napping. An explanation for this could be that sleep quality was only assessed with single item, in a yes/no format, instead of having Likert-scale response options. In an Australian study among older adults, those taking sleep medication were more likely to nap, and they also tend to nap longer than other subjects [8], while in our study among the working sample, the use of sleep medication was negatively associated with short and long napping. It should be mentioned here that the prevalence of past 2-weeks sleep medication use in the working sample of this study was 0.5% (*n* = 22) which reduces the ability to draw conclusions from this finding; in comparison in a sample of older adults in Brazil the prevalence of past 12-month sleep medication use was 20% [39]. Overall, based on a systematic review, “there is minimal evidence to indicate that napping is detrimental for older adults’ nighttime sleep.” [41].

### Examine the advantages and disadvantages of the study

The study’s strength was the use of a nationally representative sample of older adults in the Philippines, combined with standardized measures that were modified from the Health and Retirement Study. Due to self-reporting, most data might have limitations. Furthermore, since the study’s data were cross-sectional, we are unable to establish a causal relationship between any of the related factors. Future research should take into account the time, frequency and period of napping and dietary habits, which were not evaluated in this survey. Furthermore, at least on a sub-sample, future studies should assess objective sleep and napping data.

## Conclusion

Two in five older adults engaged regularly in napping and one in four older adults reported long napping duration. The results of the current study indicated that some of the same factors were linked to both short and long nap times, including high wealth status, and physical activity. Factors uniquely associated with short napping included late insomnia, and uniquely associated with long napping included older age, urban residence, not working status, ADL limitations, not poor self-rated health status, and not current alcohol use. Further unique factors with short napping among those who were working included not current smoking and not taking sleep medication and unique factors with long napping among those who were working included not having depressive symptoms and not taking sleep medication. Among those who were not working, male sex and without current alcohol use was associated with short and long napping, and older age, urban residence ADL limitations were uniquely associated with long napping. This seems to largely confirm that different factors contribute to short or long napping.

## Data Availability

The datasets generated during and/or analysed during the current study are available in the Economic Research Institute for ASEAN and East Asia, https://www.drdf.org.ph/lsahp-baseline-data-request-portal/.

## Declarations

All methods were carried out in accordance with relevant guidelines and regulations and have been performed in accordance with the Declaration of Helsinki.
